# Editorial: Physiological and molecular basis of fruit ripening and development and its application for quality improvement

**DOI:** 10.3389/fpls.2023.1138912

**Published:** 2023-01-23

**Authors:** Vasileios Ziogas, Christos Bazakos, Athanassios Molassiotis

**Affiliations:** ^1^ Laboratory of Citruculture, Institute of Olive Tree, Subtropical Plants and Viticulture, ELGO-DIMITRA, Chania, Greece; ^2^ Institute of Plant Breeding and Genetic Resources, ELGO – DIMITRA, Thessaloniki-Thermi, Greece; ^3^ Joint Laboratory of Horticulture, ELGO-DIMITRA, Thessaloniki-Thermi, Greece; ^4^ Max Planck Institute for Plant Breeding Research, Department of Comparative Development and Genetics, Carl-von-Linné-Weg 10, Cologne, Germany; ^5^ Laboratory of Pomology, Department of Horticulture, Aristotle University of Thessaloniki, Thessaloniki-Thermi, Greece

**Keywords:** breeding, fruit ripening, quality, pepper, grape, apple, tomato, molecular mechanisms

## 1 Introduction

Fresh fruits are an important agricultural commodity since they constitute a fundamental part of the dietary pyramid. Their nutritional value is unique as they are a superb source of antioxidants, fibers, and vitamins (Dorais et al., 2008). The present Research Topic aims to provide a collection of articles with novel results on fresh fruit ripening, development, and fruit quality *via* the prism of molecular and breeding mechanisms. Liu et al. review in-depth the research aspects regarding progress on the genetic basis of apple fruit quality traits. Insights into the mechanisms of genetic variation and molecular breeding that control and manipulate important nutritional quality attributes, are also given. The scientific work of Peng et al., provides novel knowledge on the molecular and physiological mechanisms that govern ripening in grapes, after the implementation of girdling as a cultivation technique. Factors such as color turning, early maturation, and sugar accumulation are shown to be directly affected by girdling, and the mechanisms of these effects are analyzed and clarified at a molecular level. The work of He et al. analyses the impact of the implementation of blue light frequencies in tomato fruits, and its direct effect on ripening and nutritional quality. The application of supplemental LED blue light can improve the ripening process and the nutritional attributes of tomato fruits. Last but not least, Lozada et al. review and elucidate aspects related to chili pepper breeding and provide an in-depth overview of the novel omics tools which can be implemented to facilitate genetic improvement.

## 2 Genetic basis of quality attributes and omics technologies

Fruit quality attributes are determined by various polygenes or oligogens (Conner et al., 1998; Zheng et al., 2020). Fruit quality is linked with appearance, taste, nutritional value, shelf-life duration, and the ability to resist transportation injuries (Chen et al., 2015). Clarification of the mechanisms and molecular cascades that participate in the development of fruit phenotypes is of paramount importance for plant breeders. In the review article by Liu et al., the authors stated that research of genetic characteristics provides an important basis for crop breeding. The authors summarized the recent knowledge related to genetic studies on various apple fruit quality attributes such as appearance, flavor, nutritional value, ripening, and storability, and provided an in-depth discussion of the mapping of Quantitative Trait Loci’s (QTLs), screening of molecular markers that can be used in breeding, and pinpointed crucial genes that are directly linked with each one of the proposed quality attributes. In their work the authors highlighted the fact that perennial fruit trees mainly exhibit several attributes of genetic variation. Most perennial fruit trees are self-incompatible, heterozygous, and are characterized by a long juvenile phase. Moreover, most of the quality traits such as fruit size, color, sugar–acid content, aroma, and polyphenol concentration are polygenic quantitative traits. Furthermore, the authors stated that through the development of omics technologies such as genomics, proteomics, metabolomics, phenomics, genome-wide association analysis (GWAS), metabolic GWAS, and structural variation analysis, great progress has been made toward the exploitation of genetic factors that govern fruit quality. The beneficial impact of the implementation of omics technologies in fruit breeding was also presented.

The article by Lozada et al. presented various omics tools that plant breeders can use to address the constraints of chili peppers. The authors presented data related to the essential need to preserve genetic biodiversity in germplasm collections. Moreover, the authors tried to demonstrate and review various tools which can be used for the genetic improvement of chili peppers. The genetic profiles of crucial complex traits in chili peppers, such as yield, resistance to pathogens, heat levels, and mechanical harvesting adaptability, can be analyzed *via* the use of mapping methods such as linkage analysis or GWAS. The proposed methods provide the ability to recognize genome loci, named QTL, that directly affect variations in the exploited phenotype. The authors also discuss how agricultural robots can be used as facilitators of mechanical harvesting in chili peppers. Various studies encourage the use of harvesting robots since they exhibit high localization, high harvesting success rates, low fruit damage, and overall performance equal to that of human harvesting.

## 3 Impact of cultivation practices on fruit quality

The scientific work of Peng et al. sheds light on the molecular and physiological mechanisms that are activated after the implementation of girdling in grapes, leading to their advanced ripening. Trunk girdling is a cultivation practice that is performed to control vegetation growth and development (Binkley et al., 2006); however, the underlying mechanism is poorly understood. It has been stated that girdling can induce phenylpropanoid metabolism and can induce the biosynthesis of phytohormones in the fruitlets of girdled vines (Tyagi et al., 2020). In their work, girdling was performed on 5-year-old ‘Summer Black” grapevines at early veraison and in-depth transcriptional and physiological analyses were performed. The authors found that this cultivation practice promotes sugar accumulation and fruit color development, while favoring ripening within 25 days. The same group also found that girdling triggers the upregulation of genes related to sugar metabolism, anthocyanin biosynthesis, ethylene biosynthesis, and phytohormone biosynthesis (abscisic acid and brassinosteroid). The authors identified a total of 120 differentially expressed transcription factors from 29 gene families, that actively participate in the regulation of grape berry development and ripening.

Regarding cultivation practices, the role of supplemental blue light application as a technique to improve the ripening and nutritional quality of fresh fruit tomatoes was presented by He et al. It is well-known that light triggers the activation of various physiological and metabolic cascades in plants (Galvão and Fankhauser, 2015) and that supplemental light is needed for proper fruit development in greenhouses and weak light conditions (Fanwoua et al., 2019). The authors showed that supplemental blue light can accelerate flower induction, favoring fruit ripening in 3–4 days in tomato plants grown in plastic greenhouses. This beneficial effect is directly linked with ethylene biosynthesis, whose promotion also facilitates fruit color change and maturation. Furthermore, the use of blue LED light also boosts antioxidant metabolism and induces the biosynthesis of antioxidant compounds such as lycopene, phenolics, flavonoids, and ascorbic acid. The implementation of this agricultural practice can provide superior nutritional attributes and sensory profiles compared with tomato plants grown in low light conditions.

## Author contributions

VZ wrote the editorial with inputs from CB and AM. All authors contributed to the article and approved the submitted version
